# Supplementary material for: Stable water splitting using photoelectrodes with a cryogelated overlayer
Source: Nat Commun. 2024 Feb 19;15:1495. doi: 10.1038/s41467-024-45701-5 (PMC10876939; doi:10.1038/s41467-024-45701-5)
Supplement: Supplementary file 1 — Supplementary Information [file 41467_2024_45701_MOESM1_ESM.pdf]

Supplementary Information for

Stable water splitting using photoelectrodes with a cryogelated overlayer

Authors: Byungjun Kang[1†], Jeiwan Tan[2,3†], Kyungmin Kim[2], Donyoung Kang[1], Hyungsoo Lee[2], Sunihl Ma[2], Young Sun Park[2], Juwon Yun[2], Soobin Lee[2], Chan Uk Lee[2], Gyumin Jang[2], Jeongyoub Lee[2], Jooho Moon[2*], Hyungsuk Lee[1*]

[1]School of Mechanical Engineering, Yonsei University, Seoul 03722, Republic of Korea

[2]Department of Materials Science and Engineering, Yonsei University, Seoul 03722, Republic of Korea

[3]Current address: Chemistry and Nanoscience Center, National Renewable Energy Laboratory, Golden, CO 80401, USA

*Corresponding author. Email: jmoon@yonsei.ac.kr (J.M.); hyungsuk@yonsei.ac.kr (H.L.)

† These authors contributed equally to this work.

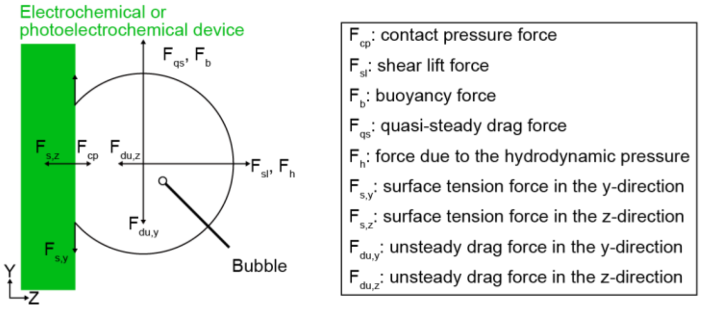

Supplementary Fig. 1. Schematic of the physical forces applied on the bubbles and the electrochemical or photoelectrochemical devices. The schematic was illustrated based on the findings in the previous studies[1,2].

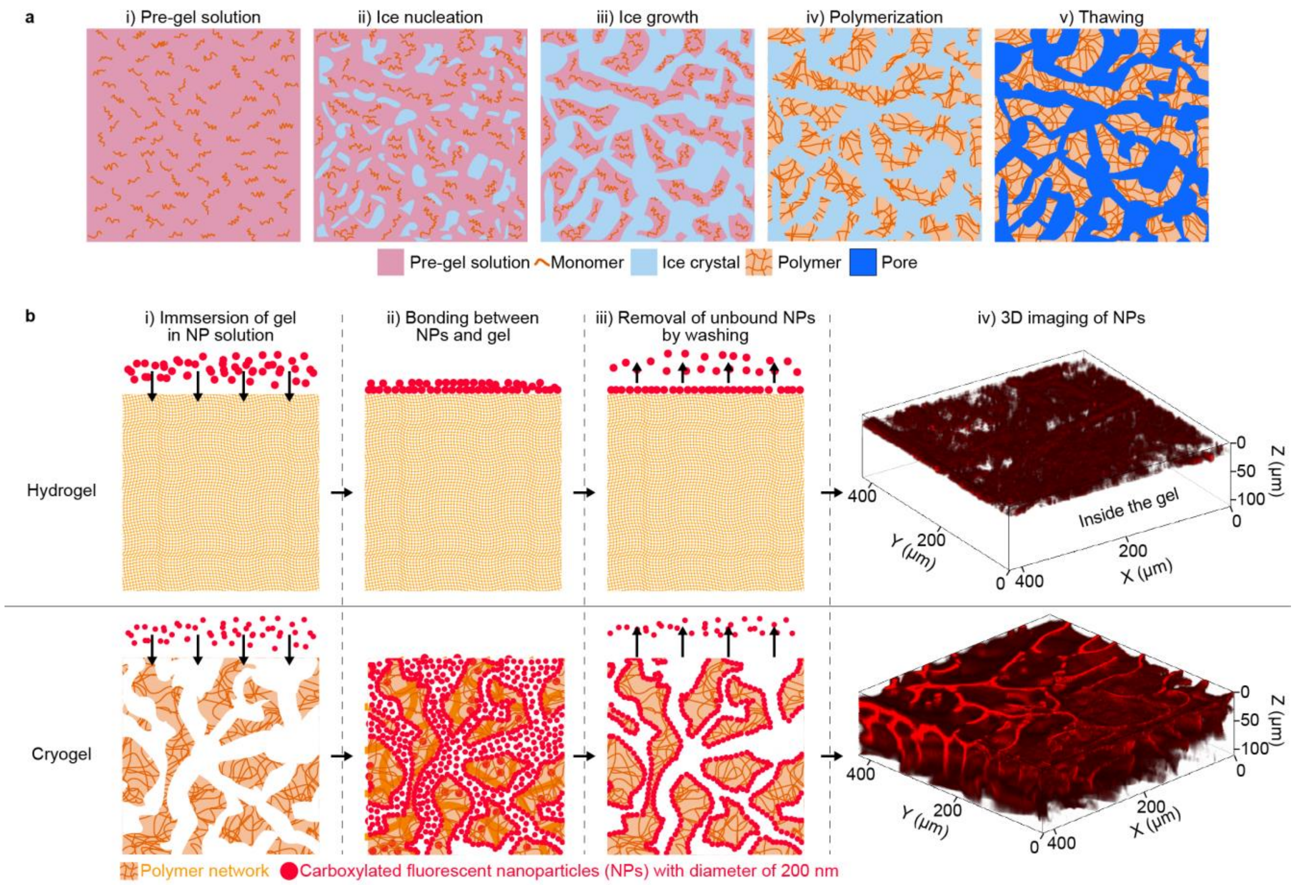

Supplementary Fig. 2. Fabrication and structural characterization of the cryogelated hydrogel overlayer. (a) Schematic of the cryogelation process. (b) Preparation of the sample for three-dimensional imaging of 200 nm nanoparticles (NPs) infiltrated into the interconnected porous structure and bound to the polymer wall. For the polyacrylamide hydrogel with an apparent pore diameter of less than 20 nanometers[3], the 200 nm NPs could not penetrate the gel sample.

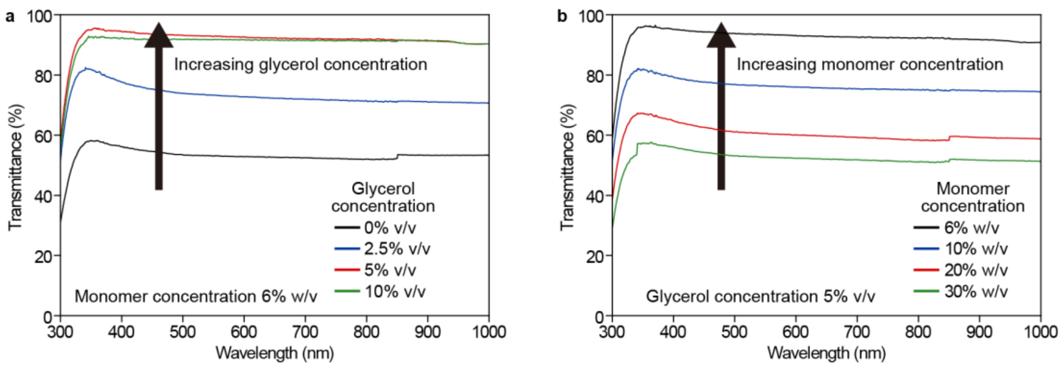

Supplementary Fig. 3. Optical transmittance of the cryogelated polyacrylamide layer as a function of (a) glycerol concentration and (b) monomer concentration in a pre-gel solution. The black, blue, red, and green solid lines in (a) represent the transmittance when the glycerol concentration was 0% v/v, 2.5% v/v, 5% v/v, and 10% v/v, respectively. The black, blue, red, and green solid lines in (b) represent the transmittance when the monomer concentration of the hydrogel was 6% w/v, 10% w/v, 20% w/v, and 30% w/v, respectively. The monomer concentration was fixed at 6% w/v (weight/volume percentage) for (a), and the glycerol concentration was fixed at 5% v/v (volume/volume percentage) for (b). The thickness of the cryogelated polyacrylamide layer was 400 μm for all samples. The cryogel utilized in the experiments of this study was fabricated using a monomer concentration of 6% w/v and glycerol concentration of 5% v/v.

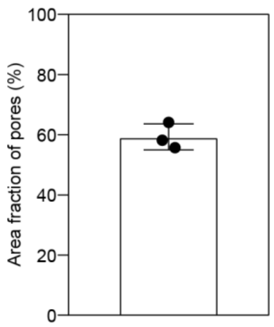

Supplementary Fig. 4. Area fraction of micropores of the cryogel overlayer obtained from the image-based analysis of the sectional image of the cryogel. The error bars represent one standard deviation (number of samples = 3).

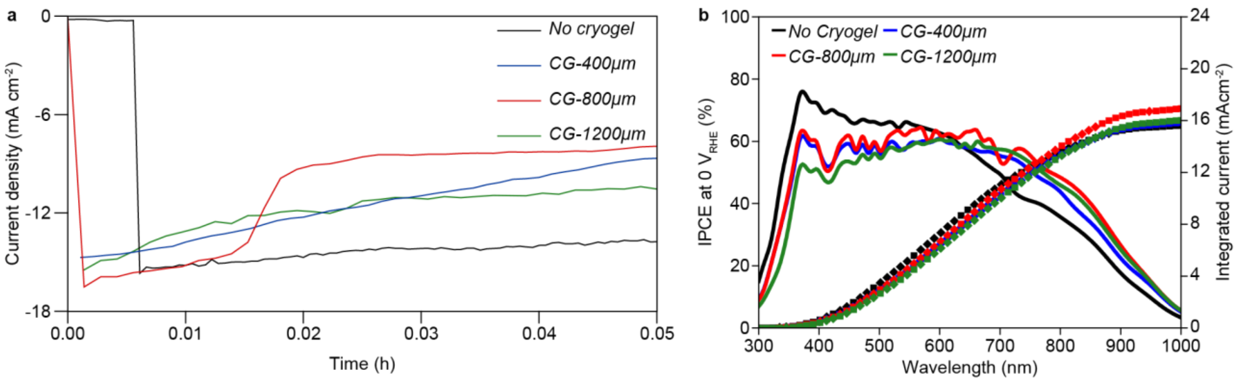

Supplementary Fig. 5. Characterization of the photoelectrochemical characteristics of Sb_2Se_3 photocathodes with and without the cryogel overlayer. (a) The current density (J_{ph})-time profile of Sb_2Se_3 photocathodes with and without the cryogelated overlayer measured via chronoamperometry at 0 V_{RHE} under 1-sun illumination during 0 to 0.05 h. The devices incorporating cryogels with thicknesses of 400 µm, 800 µm, and 1200 µm were referred to as *CG-400µm*, *CG-800µm*, and *CG-1200µm*, respectively. The device without the cryogel was denoted as *no cryogel*. The black, blue, red, and green solid lines represent the J_{ph}-time profile of *no cryogel*, *CG-400µm*, *CG-800µm*, and *CG-1200µm*, respectively. (b) The incident photon-to-current conversion efficiency (IPCE) spectra at 0 V_{RHE} (solid line) and integrated current (dotted line) for *no cryogel* (black), *CG-400µm* (blue), *CG-800µm* (red), and *CG-1200µm* (green). The integrated IPCE values for all devices using the solar AM 1.5 spectrum were similar to the initial values obtained prior to J_{ph} drop. The integrated IPCE value, calculated by integrating the values across all wavelength ranges, did not significantly change by the cryogel coating. For *CG-400µm*, *CG-800µm*, and *CG-1200µm*, the IPCE characterization was conducted at the freshly made state before the initial J_{ph} drop occurred.

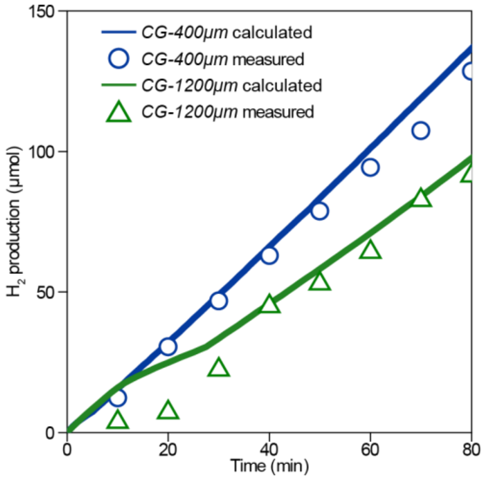

Supplementary Fig. 6. Hydrogen production as a function of operation time during the stability test for the devices with 400 µm-thick (*CG-400µm*; blue) and 1200 µm-thick (*CG-1200µm*; green) cryogel overlayers. The solid line represents the calculated amount of gas produced by the devices, assuming 100% Faradaic efficiency, based on the measured current for each device. The symbols indicate the amount of gas production determined through experimental gas chromatography analysis.

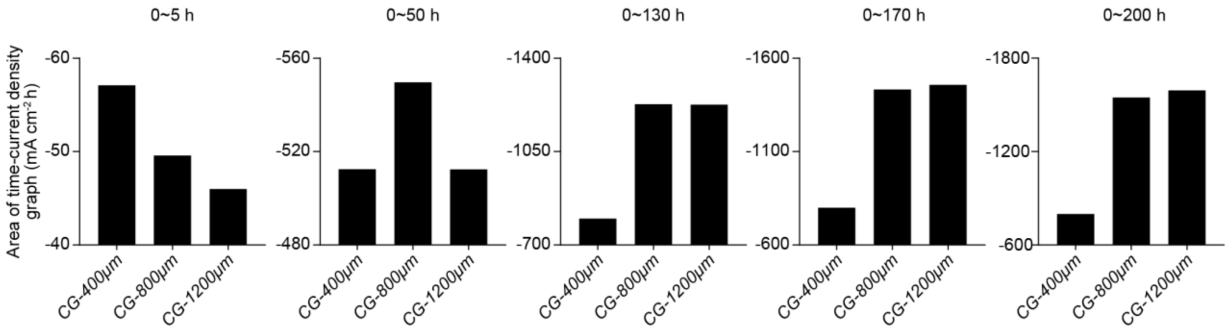

Supplementary Fig. 7. Area of the current density-time graph of *CG-400µm*, *CG-800µm*, and *CG-1200µm* for the duration of 0~5 h, 0~50 h, 0~130 h, 0~170 h, and 0~200 h. The device coated with the cryogel having thicknesses of 400 µm, 800 µm, and 1200 µm were denoted as *CG-400µm*, *CG-800µm*, and *CG-1200µm*, respectively. Assuming that 100% of the current in the device involves the Faraday reaction, the area can indicate the relative total gas production efficiency.

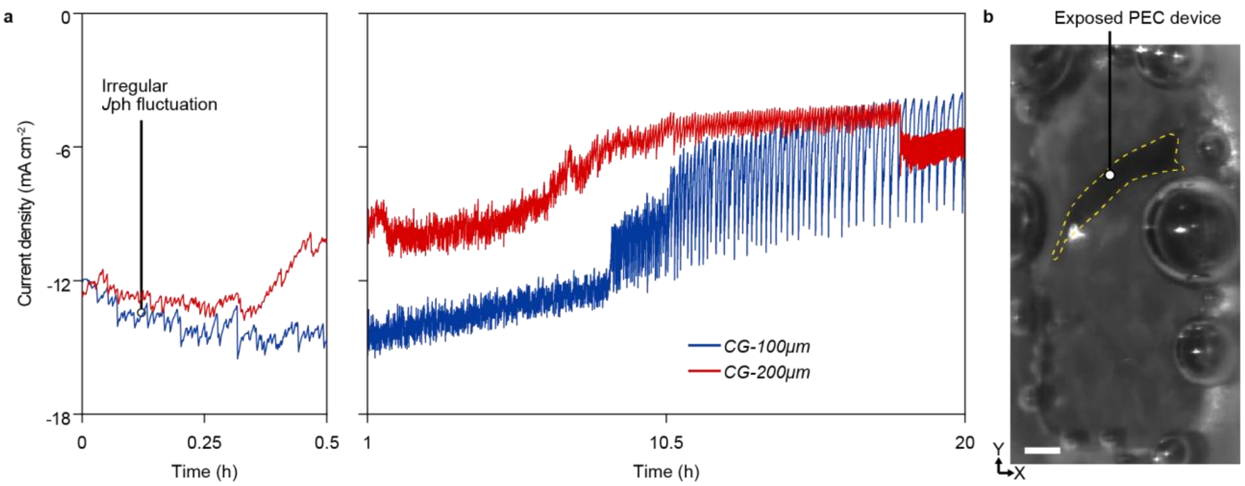

Supplementary Fig. 8. Effect of a thin cryogelated overlayer on the photoelectrochemical (PEC) operation of Sb_2Se_3 photocathodes. (a) Photocurrent density (J_{ph})-time profile of the PEC device coated with 100 µm-thick and 200 µm-thick cryogel, referred to as *CG-100µm* and *CG-200µm*, respectively, measured via chronoamperometry at 0 V_{RHE} under 1-sun illumination. The blue and red solid lines represent the J_{ph}-time profile of *CG-100µm* and *CG-200µm*, respectively. (b) Photograph of the delaminated 200 µm-thick cryogelated overlayer from the PEC device after 5 min of PEC operation. The orange dotted lines denotes the region of the exposed PEC device. The scale bar represents 0.5 mm.

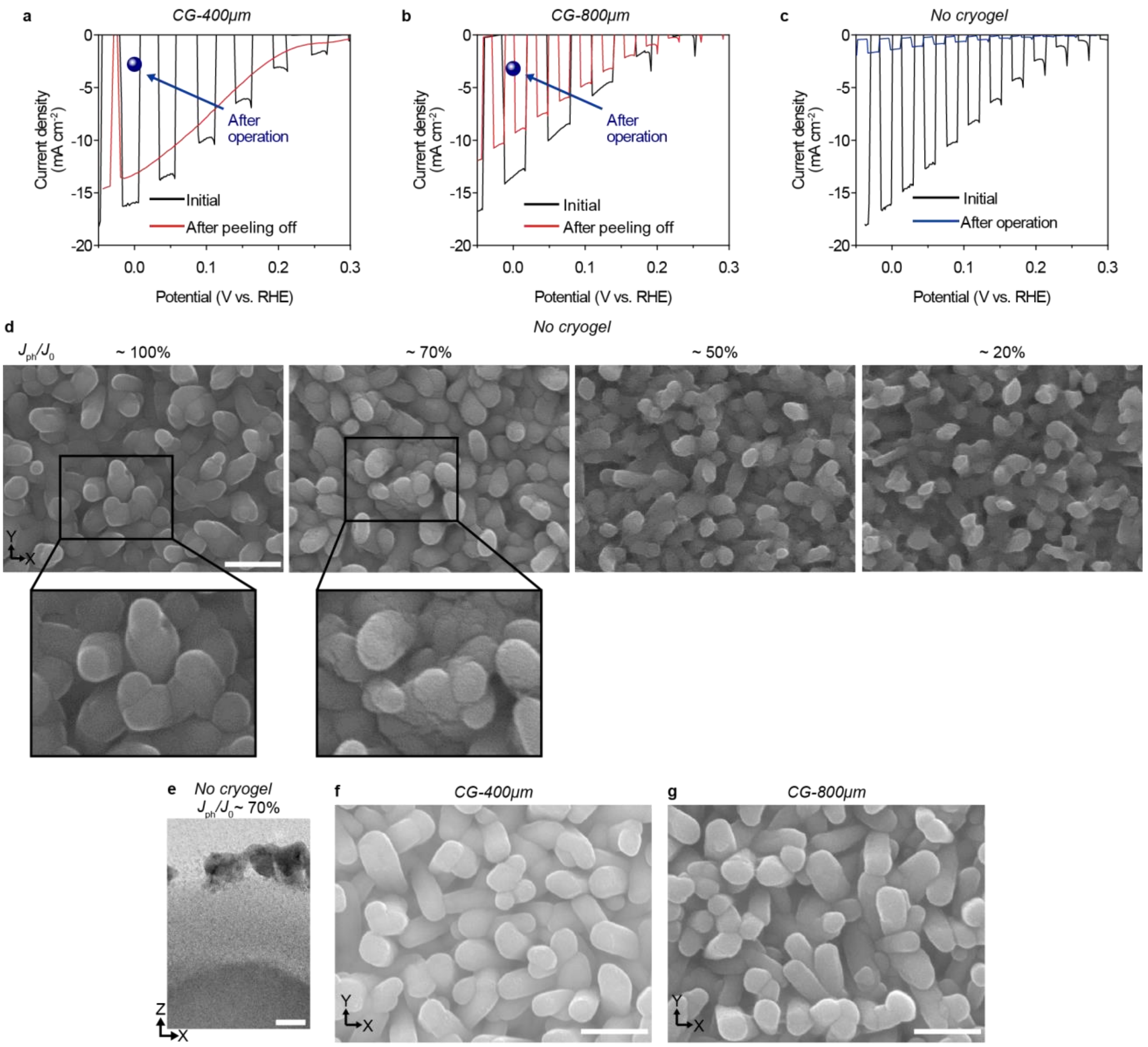

Supplementary Fig. 9. Preservation of the photoelectrochemical performance and surface morphology of the photoelectrochemical (PEC) device by the cryogelated overlayer. Photocurrent density (J_{ph}) recovery test after the overlayer was mechanically peeled off from the device. The device without the cryogel and with the cryogel overlayers with thickness of 400 μm and 800 μm were denoted as *no cryogel*, *CG-400μm*, and *CG-800μm*, respectively. The J_{ph}-potential curve of (a) *CG-400μm*, (b) *CG-800μm*, and (c) *no cryogel* measured via linear sweep voltammetry at the initial state (black), after the operation (blue), and after the mechanical peeling-off of the cryogelated overlayer (red). Morphological analysis of Sb_2Se_3 photocathodes with and without the cryogelated overlayer. (d) Scanning electron microscopy (SEM) images of the device surface of *no cryogel* when J_{ph}/J_0 was 100%, 70%, 50%, and 20%. (e) Cross-sectional transmission electron microscopy image of *no cryogel* when J_{ph}/J_0 was 70%. SEM images of (f) *CG-400μm* and (g) *CG-800μm* captured after peeling off the overlayer. The scale bars in (d), (f), and (g) represent 500 nm. The scale bar in (e) represents 10 nm. The SEM images in (d) were obtained from our previous study[3].

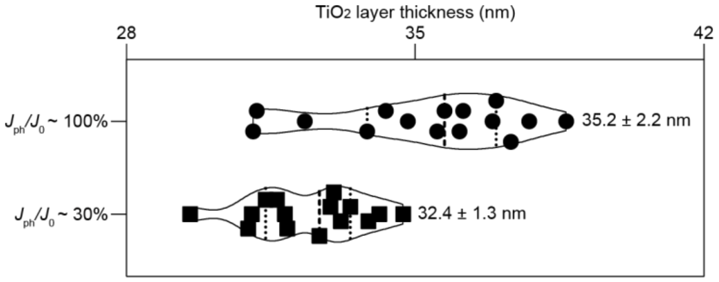

Supplementary Fig. 10. The thickness of the TiO_2 layer in the as-prepared Pt/TiO_2/Sb_2Se_3 device with a J_{ph}/J_0 of 100% and the *CG-1200μm* at a J_{ph}/J_0 of 30%. J_{ph} represents the photocurrent density of the device, while J_0 represents its initial value. The thickness of TiO_2 layer was manually characterized from the STEM-EDS element mapping data for Ti.

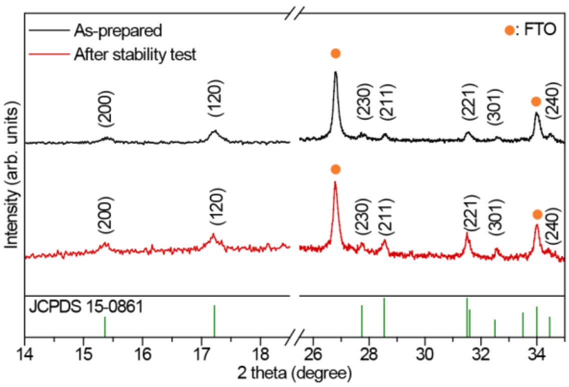

Supplementary Fig. 11. X-ray diffraction (XRD) spectra of Pt/TiO_2/Sb_2Se_3 device before (black solid line) and after the stability test (red solid line). The crystallinity and orientation of Sb_2Se_3 were maintained, and no secondary phases were observed after the PEC operation over 200 h.

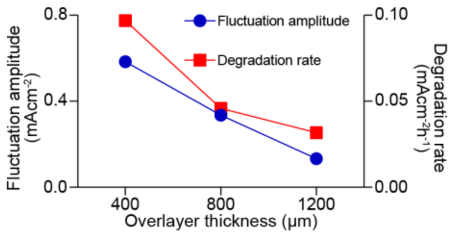

Supplementary Fig. 12. Characterization of the amplitude of the photocurrent density (J_{ph}) fluctuation (blue circle) and the rate of J_{ph} degradation (red rectangle) of the device with the cryogel overlayer having a thickness of 400 µm (*CG-400µm*), 800 µm (*CG-800µm*), and 1200 µm (*CG-1200µm*). The fluctuation amplitude of J_{ph} was obtained by one dimensional fast Fourier transform of J_{ph} for 5.5–6.0 h. The J_{ph} degradation rate was obtained by fitting the J_{ph}-time curve using linear regression. The duration of the J_{ph}-time curve ranged from 1–120 h for *CG-400µm* and 1–210 h for *CG-800µm* and *CG-1200µm.*

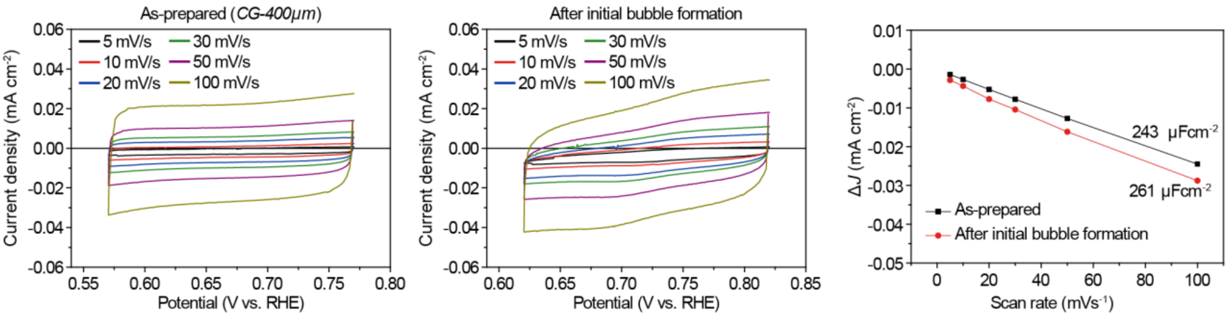

Supplementary Fig. 13. The electrochemical surface area (ECSA) analysis using cyclic voltammetry before and after the formation of the initial bubble. The double layer capacitance E_{dl} of *CG-400μm* before and after the bubble formation was similar. The ECSA can be calculated by dividing E_{dl} by a specific capacitance determined by the electrodes.

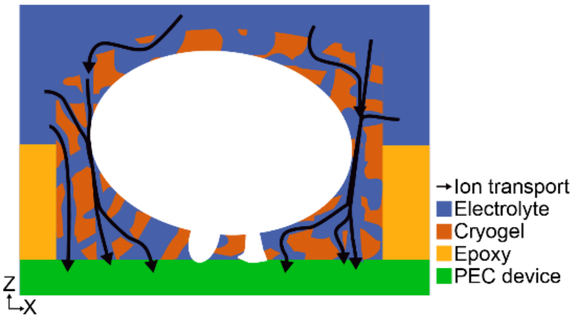

Supplementary Fig. 14. Schematic of the ion transport through the cryogel containing the trapped bubble.

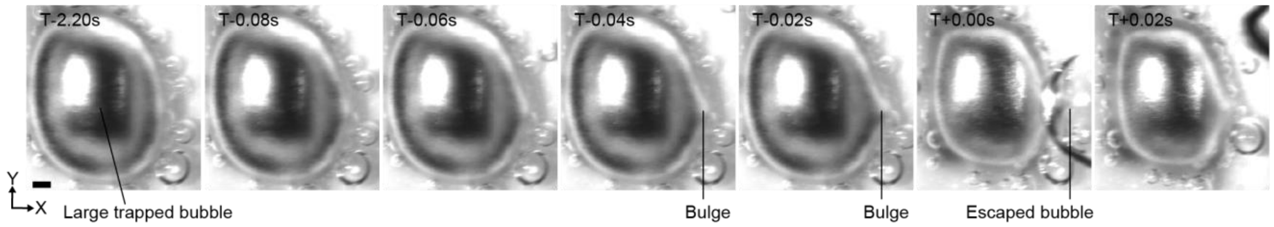

Supplementary Fig. 15. Sequential images of the bubbles right before and after the bulge formation for *CG-800 μm* shown in Figure 2a. The time point of the bubble escape is denoted as 'T.' The scale bar represents 0.5 mm.

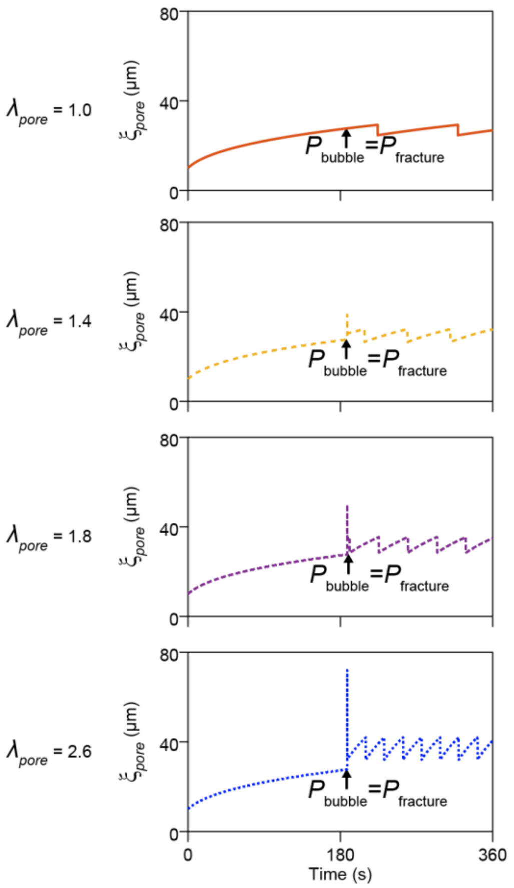

Supplementary Fig. 16. Theoretical analysis of the pore size ξ_{pore} at various degree of pore coalescence λ_{pore}.

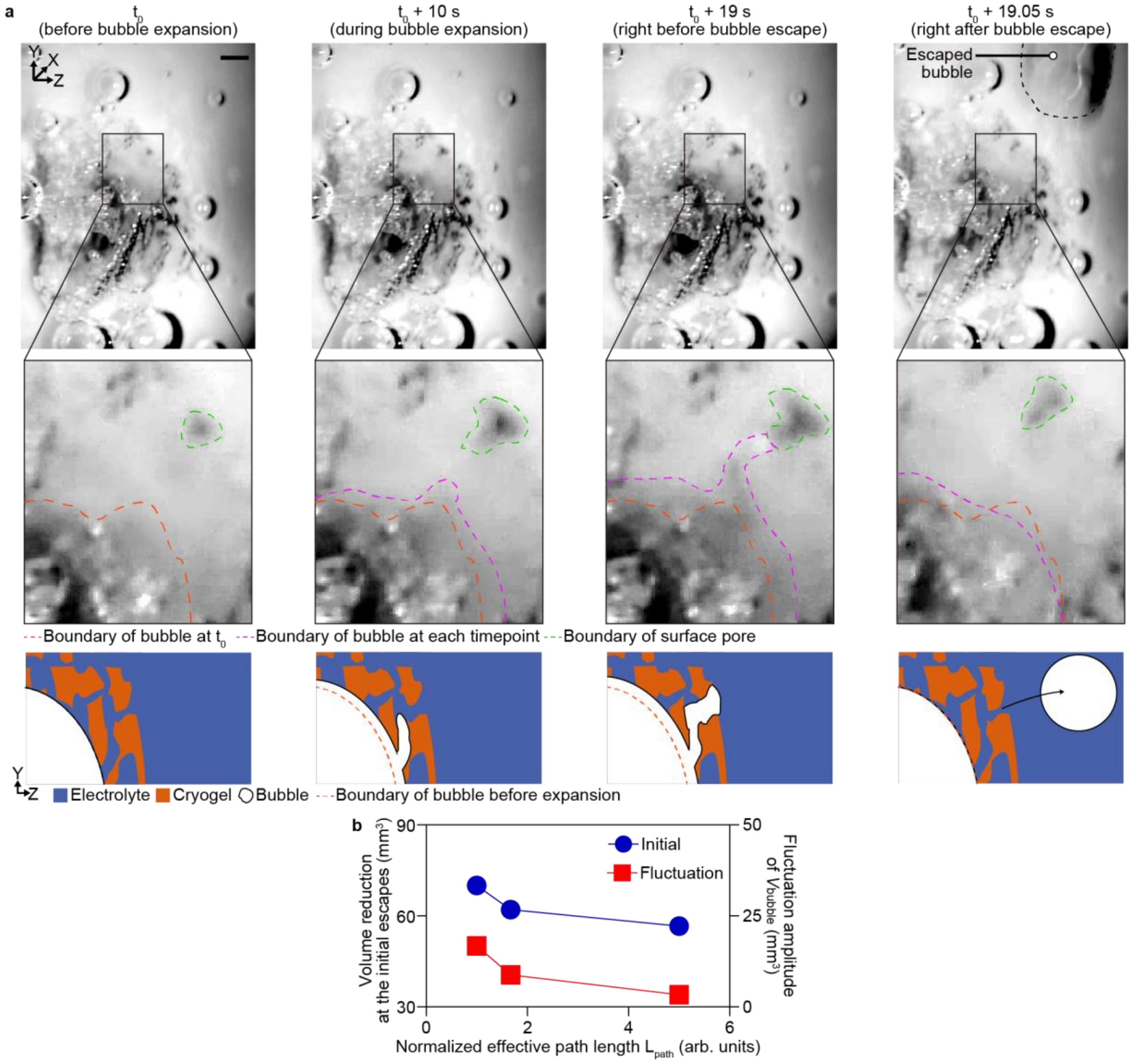

Supplementary Fig. 17. Gas transport through the layer of micropores between the trapped bubble and the overlayer surface. (a) Photographs and schematic of the bubbles in the device with the 1200 μm-thick cryogel *CG-1200μm* during the regular fluctuation of the J_{ph} at ~30 h. The orange and magenta dotted lines represent the boundary of the bubbles at the timepoint of the first image and at each timepoint, respectively. The green dotted lines indicate the boundary of the surface pores. The scale bar represents 1 mm. (b) Volume reduction at the initial bubble escape (blue circle) and fluctuation amplitude of the bubble volume V_{bubble} (red rectangle) as a function of the normalized effective path length for the gas transport L_{path}.

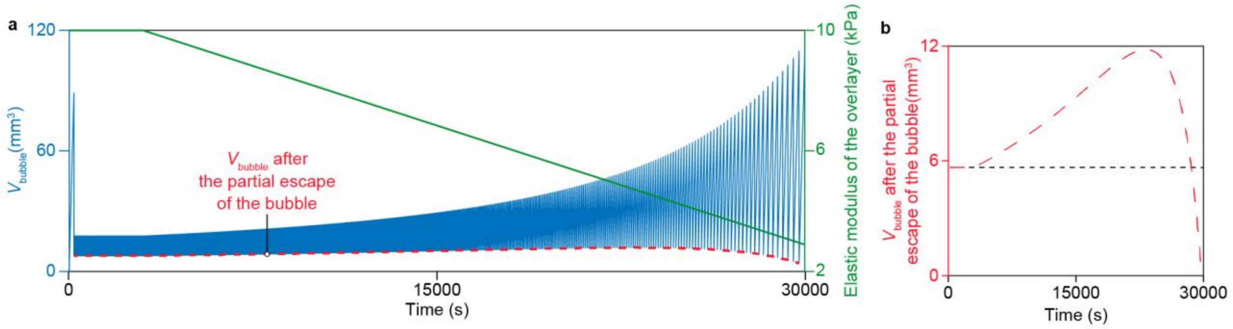

Supplementary Fig. 18. Effect of overlayer softening on the dynamics during cycles of bubble expansion and escape. (a) Theoretical analysis of the volume of the trapped bubble V_{bubble} (left; blue solid line) during reduction of the elastic modulus of the overlayer over time (right; green solid line). (b) Theoretical analysis of V_{bubble} after the partial escape of the bubble over time (red dotted line).

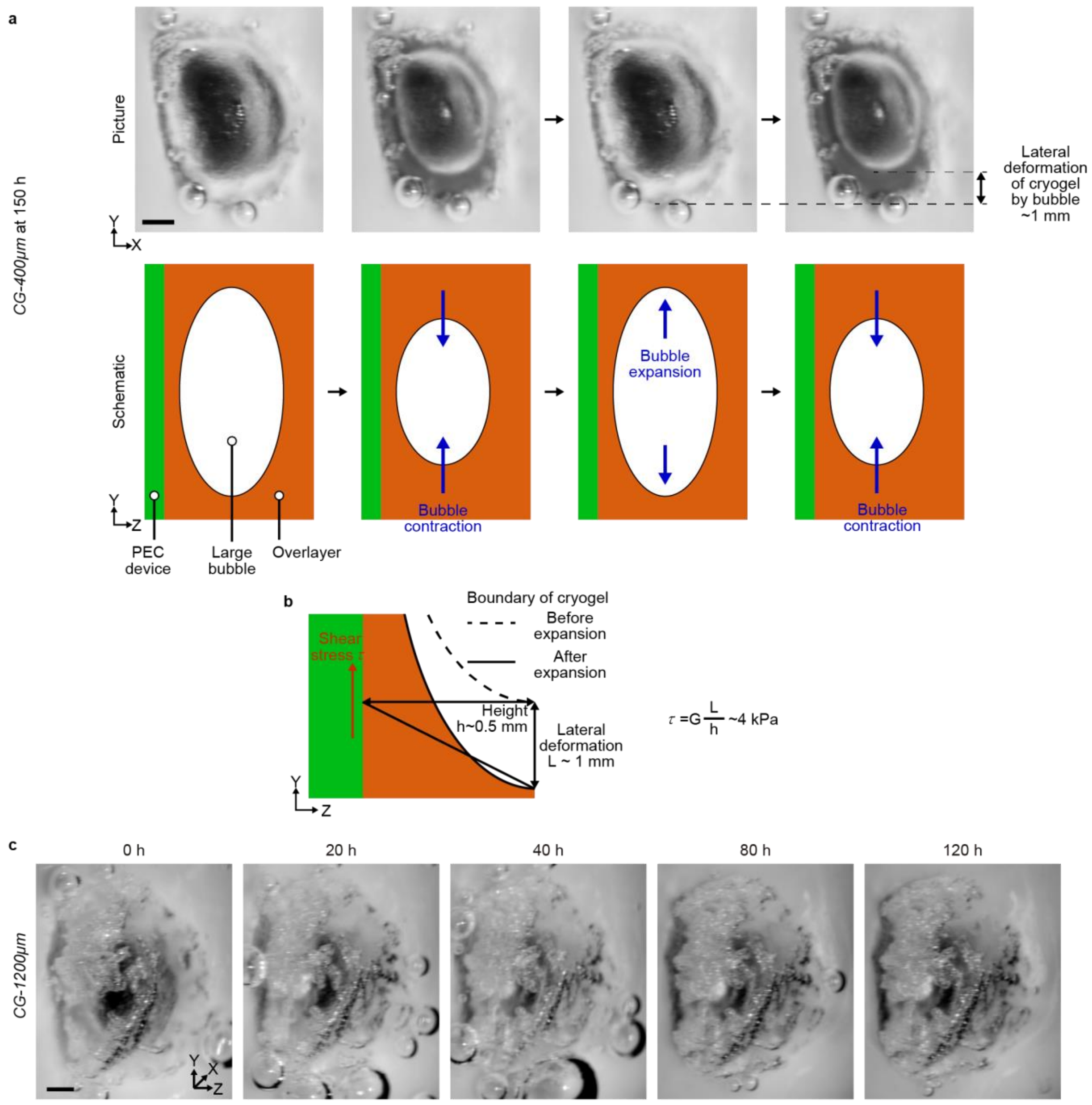

Supplementary Fig. 19. Long-term observation of bubbles in the cryogelated overlayer. (a) Photographs and cross-sectional schematic of the contraction and expansion of bubbles in the device coated with 400 µm-thick cryogel (*CG-400µm*) at 150 h. At 150 h, the trapped bubble contracted and expanded not only in the direction of the overlayer thickness but also in the direction parallel to the device surface. (b) Theoretical estimation of the shear stress applied on the device surface by the bubble expansion. In the calculation, the shear modulus was assumed to be 2 kPa obtained from the polyacrylamide hydrogel having the elastic modulus of 7 kPa[4]. (c) Photographs of the bubbles in the device with the cryogel overlayer having a thickness of 1200 µm (*CG-1200µm*) at 0, 20, 40, 80, and 120 h. The scale bars represent 1 mm.

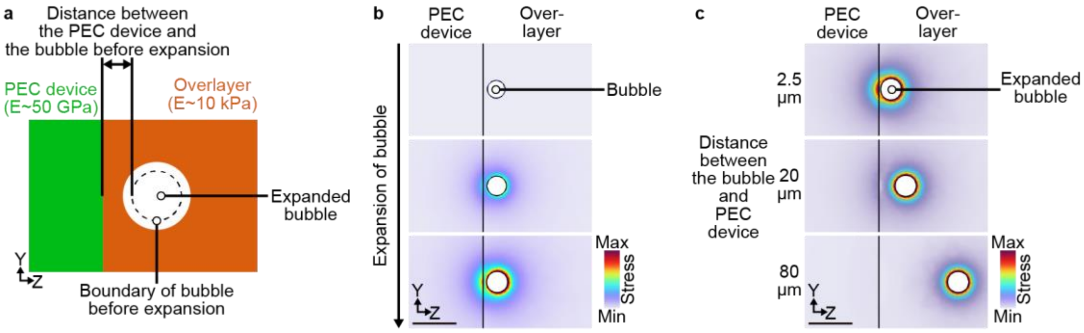

Supplementary Fig. 20. Numerical analysis of the mechanical stress applied on the photoelectrochemical (PEC) device by the expansion of bubble inside the cryogel overlayer. (a) Schematic of the numerical simulation. (b) Finite-element method (FEM) results of the mechanical stress applied on the overlayer and PEC device during the expansion of bubble confined in the overlayer. (c) FEM result of the mechanical stress on the overlayer and PEC device with varying distance between the bubble and PEC device. The scale bars represent 50 μm. The elastic modulus of the PEC device and overlayer were assumed to be 50 GPa and 10 kPa, respectively.

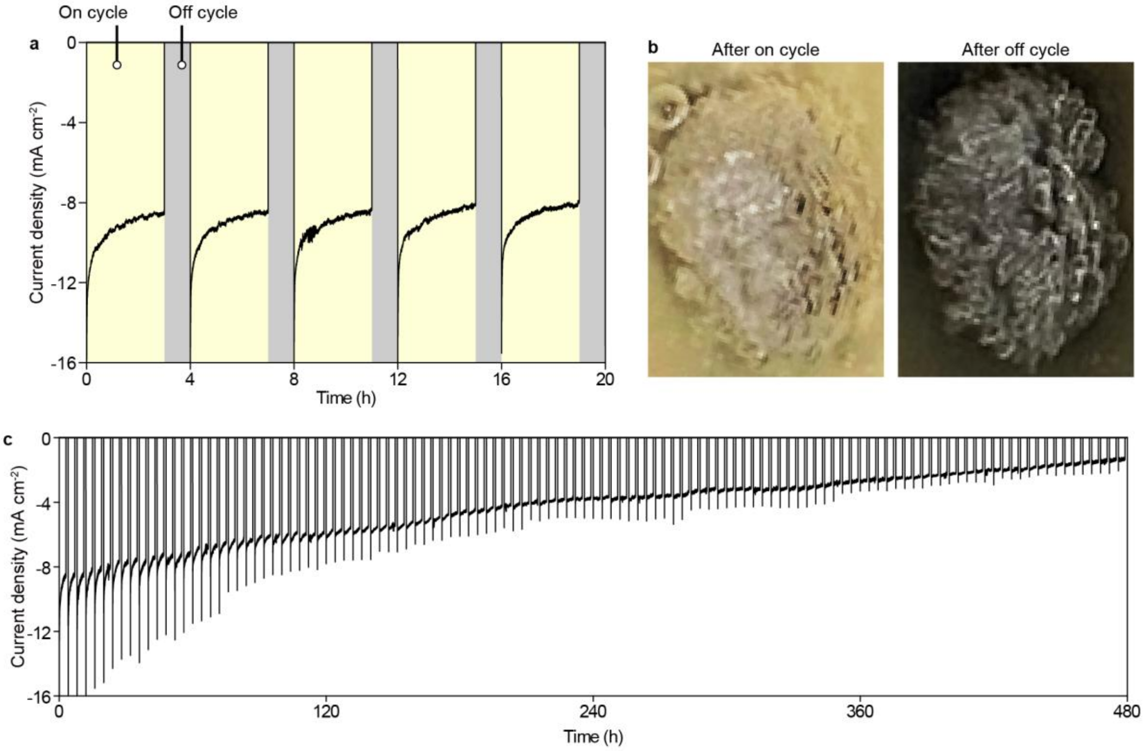

Supplementary Fig. 21. On/off cycle operation of a Sb_2Se_3 photocathode covered with the cryogelated overlayer with interconnected micropores. (a) The photocurrent density-time profile of *CG-1200μm* operated by on (yellow region) and off cycles (gray region) during 20 h. The duration of the on and off cycles was 3 h and 1 h, respectively. (b) Pictures of the bubbles in *CG-1200μm* after the 'on cycle' and 'off cycle'. (c) The photocurrent density-time profile of *CG-1200μm* operated by on and off cycles for 480 h.

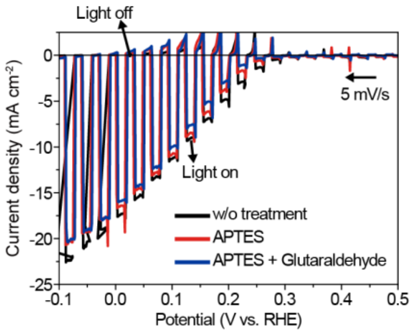

Supplementary Fig. 22. The effect of self-assembled monolayer (SAM) treatment on the photoelectrochemical (PEC) performance. The current density J_{ph}-potential curve of $Pt/TiO_2/Sb_2Se_3$ devices without (w/o) treatment (as-prepared; black), after the APTES treatment step (red), and after the glutaraldehyde treatment step (blue). The SAM treatment did not significantly alter the PEC performance.

No.	Device components	Duration	Reference
1	$Pt/TiO_2/Sb_2Se_3$	2 h	[5]
2	$Pt/TiO_2/AZO/Cu_2O$	20 min	[6]
3	$Pt/TiO_2/Ga_2O_3/Cu_2O$	2 h	[7]
4	$Pt/TiO_2/CdS/CuO$	30 min	[8]
5	$Pt/TiO_2/CdS/CZTS$	1 h	[9]

Supplementary Table 1. Device components and operation duration photocathodes composed of Pt catalyst, TiO_2 layer, and low-cost thin-film light absorber.

Supplementary Note 1. Theoretical analysis of the bubble escape from the cryogelated overlayer

In a porous membrane, bubbles can escape through a pore when the pressure of the bubble is higher than the capillary pressure P_{cap}, which is determined by the Laplace–Young equation as

$$P_{cap} = \frac{2\gamma}{\xi_{pore}} \quad (1)$$

, where γ and ξ_{pore} represent the surface tension coefficient and pore radius, respectively[10].

The size of the pores on the surface of cryogelated overlayer and the corresponding capillary pressure can be significantly varied by the overlayer deformation during bubble expansion and by the pore coalescence during the local fracture in the overlayer.

To understand the physical mechanism of the bubble dynamics in the cryogelated overlayer, we analyzed the pressure and volume of the bubble, pore size, and corresponding capillary pressure for cases without and with the pore coalescence by the overlayer fracture.

It was assumed that the gas molecules produced by the PEC device were fully absorbed by the large bubble trapped in the overlayer with an initial radius $r_{bubble}(t_{init})$ where t_{init} denotes the initial time point. The volume rate of gas production by the PEC device $\dot{V}_{gas}$ is set to a constant value. Thus, the volume of the trapped bubble V_{bubble} at time t increases as follows:

$$V_{bubble}(t) = V_{bubble}(t\text{-}t_{step}) + t_{step} \times \dot{V}_{gas} \quad (2)$$

where t_{step} denotes the time step. The expansion ratio $\lambda_{bubble}(t)$ for a bubble with volume $V_{bubble}(t)$ is calculated as follows[11,12]:

$$\lambda_{bubble}(t) = \frac{r_{bubble}(t)}{r_{bubble}(t_{init})} = \left(\frac{V_{bubble}(t)}{V_{bubble}(t_{init})}\right)^{1/3} \quad (3)$$

The radius of the pore on the overlayer surface was assumed to be linearly proportional to the radius of the large bubble trapped in the overlayer because the distance between two points on the surface of a sphere is linearly proportional to the radius of the sphere. The size of the pores near the surface of the overlayer ξ_{pore} at time t can be reversibly increased by bubble expansion as:

$$\xi_{pore}(t) = \frac{r_{bubble}(t)}{r_{bubble}(t_{init})}\xi_{pore,base}(t) = \lambda_{bubble}(t) \times \xi_{pore,base}(t) \quad (4)$$

where $\xi_{pore,base}$ is the pore size without the pore enlargement by the bubble expansion.

The pressure applied on the overlayer by bubble P_{bubble} can be calculated by summing the pressure generated during the nucleation of the bubble P_0 and the pressure caused by the expansion of bubbles trapped in the overlayer P_{exp} as

$$P_{bubble}(t) = P_0 + P_{exp}(t) \quad (5)$$

P_{exp} is generated due to the elasticity of the overlayer as follows:

$$P_{exp}(t) = E_{overlayer}(\frac{5}{6} - \frac{2}{3}(\lambda_{bubble}(t))^{-1} - \frac{1}{6}(\lambda_{bubble}(t))^{-4}) \quad (6)$$

where $E_{overlayer}$ is Young's modulus of the overlayer[11,12]. In the case of softening of the cryogelated overlayer, $E_{overlayer}$ is set to decrease linearly with time.

The partial volume of the bubble escapes through the surface pore of the cryogelated overlayer when $P_{bubble}(t)$ becomes larger than the capillary pressure $P_{cap}(t)$, which is calculated as follows:

$$P_{cap}(t) = \frac{2\gamma}{\xi_{pore}(t)} = \frac{2\gamma}{\xi_{pore}(t_{init})} \times \frac{\xi_{pore}(t_{init})}{\xi_{pore}(t)} = P_{cap}(t_{init}) \times \frac{\xi_{pore}(t_{init})}{\xi_{pore}(t)} \quad (7)$$

When no fracture occurs, $\xi_{pore,base}(t)$ is calculated using the following equation:

$$\xi_{pore,base}(t) = \xi_{pore,base}(t\text{-}t_{step}) \quad (8)$$

When $P_{bubble}(t)$ is higher than the critical value $P_{fracture}$, the pores at the overlayer surface can be coalesced by the local fracture. Thus, $\xi_{pore,base}(t)$ is set to increase as follows:

$$\xi_{pore,base}(t) = \lambda_{pore} \times \xi_{pore,base}(t\text{-}t_{step}) \quad (9)$$

, where λ_{pore} represents the degree of pore coalescence caused by fracturing. A higher value of λ_{pore} indicates that a larger pore is formed by the overlayer fracture.

The flow rate of the escaping air from the deflating rubber balloon can depend on the volume and pressure of the air and the radius of the inlet of balloon[13]. We assumed that the volume of the bubble escaping from the pore V_{escape} is a function of the volume and pressure of the bubble and the pore size.

In addition to the fractured pore, the trapped gas bubble should pass through the internal micropores as observed in **Supplementary Fig. 17a**. The gas transport rate is decreased by the path length which is increased by the cryogel thickness due to the resistance by the internal pores of the cryogel according to Poiseuille' Law[14]. The elastic deformation of micropores can also increase the resistance for the gas transport. Therefore, the effective path length would be determined by not only the geometry of the pores but the elastic properties of the cryogel.

The V_{escape} is calculated as follows:

$$V_{escape} = (V_{bubble,init} \times A_0) \times \left(\frac{V_{bubble}}{V_{bubble,init}}\right)^{\alpha} \times \left(\frac{P_{bubble}}{P_{bubble,init}}\right)^{\beta} \times \left(\frac{\xi_{pore}}{\xi_{pore,init}}\right)^{\delta} \times (\frac{1}{L_{path}})^{\varepsilon} \quad (10)$$

, where A_0, α, β, δ, and ε are fitting parameters. L_{path} is a normalized effective path length.

Consequently, the volume of the bubble immediately after partial escape is as follows:

$$V_{bubble}(t) = V_{bubble}(t\text{-}t_{step}) + t_{step} \times \dot{V}_{gas} - V_{escape} \quad (11)$$

A theoretical analysis of bubble expansion and escape from the overlayer is summarized in **Supplementary Fig. 23**. The parameters utilized in the theoretical analysis were determined based on the experimental observations listed in **Supplementary Table 2**.

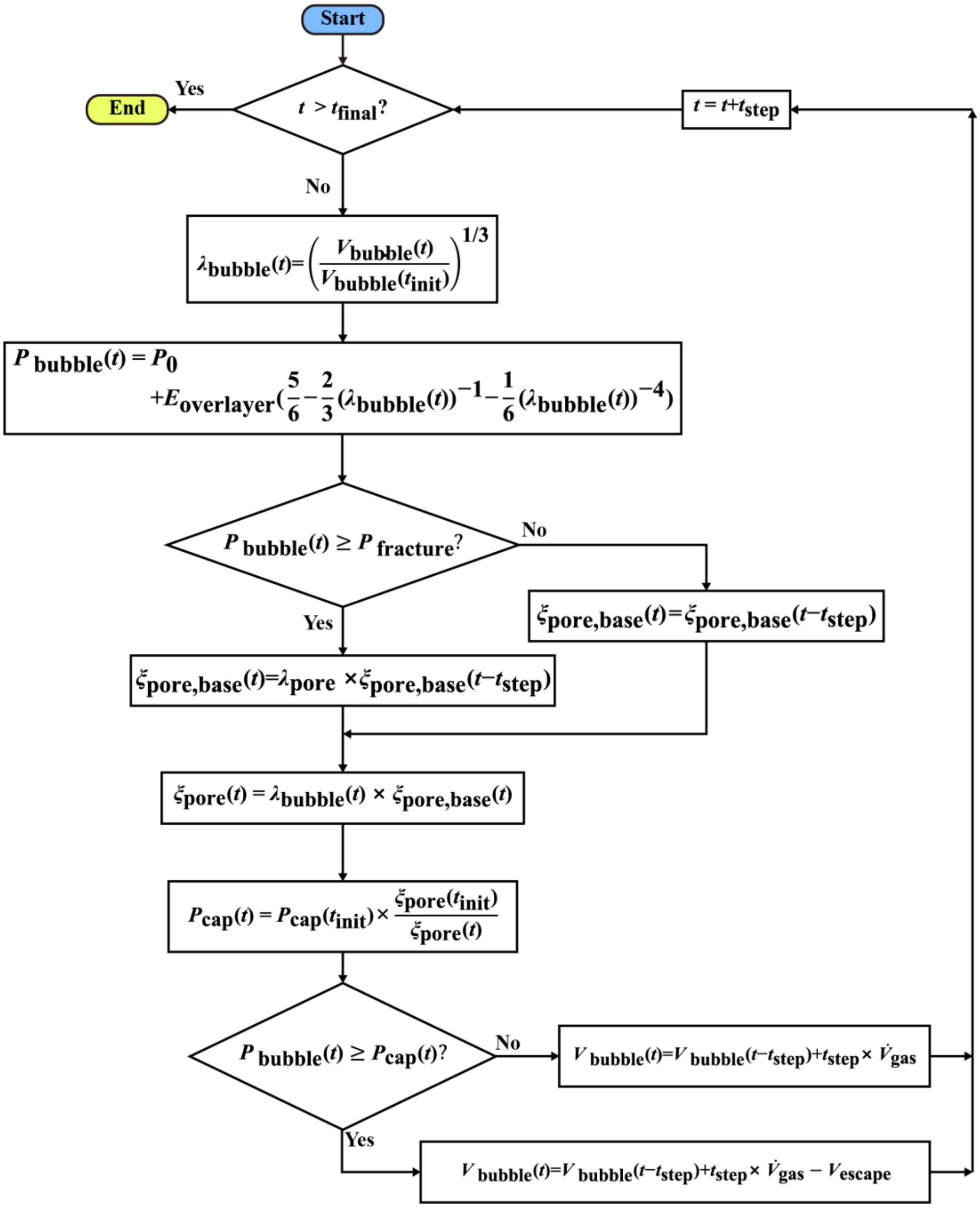

Supplementary Fig. 23. Flowchart of the theoretical analysis of the bubble escape from the pores of the cryogelated overlayer.

Symbol	Value
$\dot{V}_{\mathrm{gas}}$	0.45 $\mathrm{mm^3/s}$
t_{step}	0.01 s
$r_{\mathrm{bubble}}(t_{\mathrm{init}})$	1 mm
P_0	800 Pa
$E_{\mathrm{overlayer}}$	10 kPa
$\xi_{\mathrm{pore,base}}(t_{\mathrm{init}})$	10 µm
$P_{\mathrm{cap}}(t_{\mathrm{init}})$	20 kPa
P_{fracture}	6.6963 kPa
A_0	0.1512
α	1.0
β	0.01
δ	0.9
ε	1.0

Supplementary Table 2. Values of the parameters utilized in the theoretical analysis of the bubble escape from the pores.

Supplementary Note 2. Fabrication of photoelectrode having cryogel protector with varied internal structure

We first changed the freezing duration of the cryogelation of the device with cryogel protector having a thickness of 400 μm from 24 to 12, 48, and 72 h. When the freezing duration was 12 h, micropores were detected from the transmitted light microscopy image but the 200 nm NPs could not penetrate the micropores, implying that these micropores were disconnected (**Supplementary Fig. 24a**). The ice crystals were expected to melt before they became interconnected. The PEC device with a 400 μm-thick cryogelated overlayer and a freezing duration of 12 h was denoted as *disconnected micropores*. Because the extension of the freezing duration over 24 h did not enlarge the micropores (**Supplementary Fig. 24b and 24c**), we attempted to form additional large pores in the overlayer by using an ice-decorated cover glass instead of a flat cover glass. By placing an ice-decorated cover glass near the top boundary during the cryogelation process, interconnected micropores as well as additional macropores formed at the top surface of the cryogelated overlayer (**Supplementary Fig. 24d**). The device with a 400 μm-thick cryogelated overlayer fabricated using the ice-decorated cover is denoted as *surface macropores*. The detailed fabrication method is summarized in **Supplementary Fig. 25**.

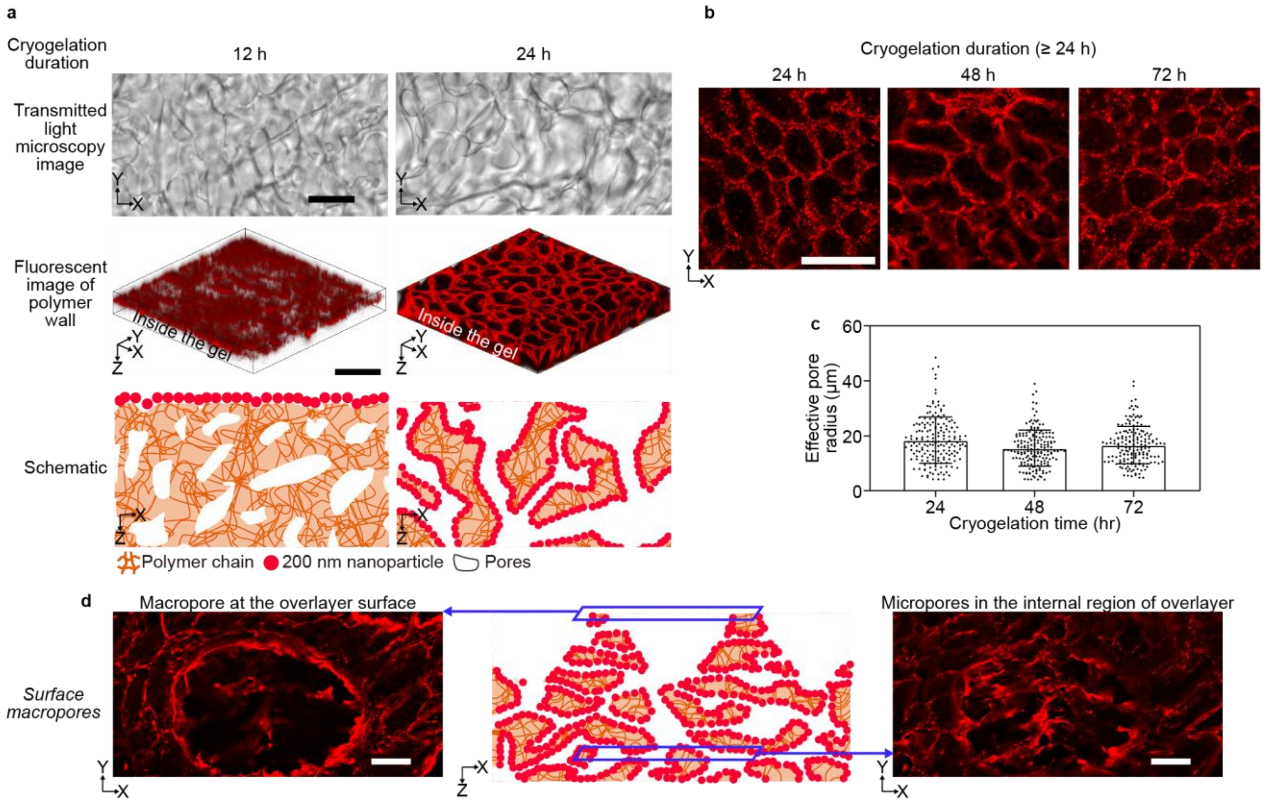

Supplementary Fig. 24. Characterization of the cryogelated hydrogel overlayer for the *disconnected micropores* and *surface macropores* samples. Image-based analysis of the internal structure of an overlayer with various porous structures. (a) Transmittance light microscopy images, representative fluorescent images, and schematic of the overlayer for cryogelation durations of 12 and 24 h. (b) Representative fluorescent images of the porous structure and (c) effective pore radius of the cryogelated sample with cryogelation durations of 24, 48, and 72 h. The error bars represent one standard deviation (n = 173 for each data point). (d) Representative fluorescent images and schematic of the cryogelated overlayer with interconnected micropores and surface macropores. The scale bars represent 100 µm.

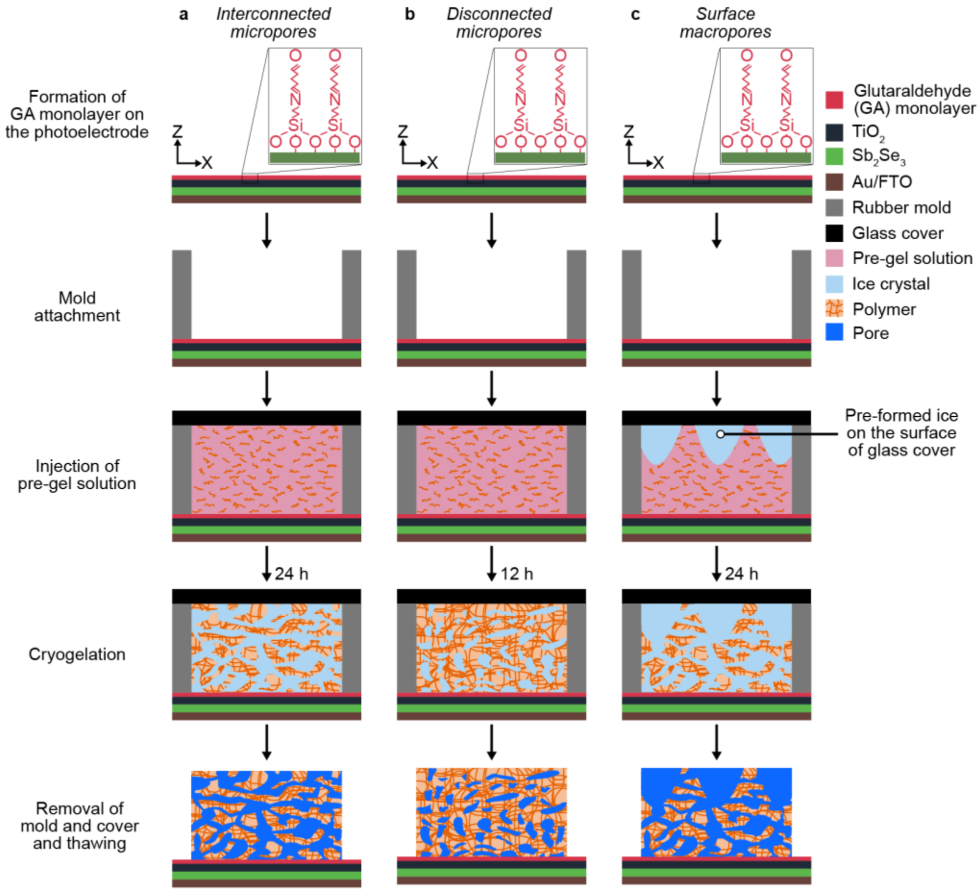

Supplementary Fig. 25. Fabrication process of devices with (a) *interconnected micropores*, (b) *disconnected micropores*, and (c) *surface macropores*.

Supplementary Note 3. PEC operation of cryogel-coated PEC devices under daily day/night cycling

Most stability tests reported so far have been conducted under continuous illumination, while practical PEC devices are subjected to daily day/night cycling. From this viewpoint, we conducted a stability test of *CG-1200μm* by performing chronoamperometry at 0 V_{RHE} with 1-sun illumination for 12 h (day cycle) and neglected the device under open-circuit potential in the dark for another 12 h (night cycle) (**Supplementary Fig. 26a**). After the initial drop and recovery, the photocurrent density J_{ph} was maintained at ~ 7 mA cm^{-2} for the first daily cycle. However, J_{ph} instantly reduced to ~ 4 mA cm^{-2} after the first night cycle and the J_{ph} degradation rate was even higher compared to the counterpart of continuous illumination upon repeated daily cycles. Based on potential monitoring during the daily night cycle (**Supplementary Fig. 26b**), we presume that the instant change of from a negative to a positive dark potential is a result of an interface destruction of the PEC device. Nevertheless, it was observed that the microbubbles accumulated in the micropores could be removed by 'diffusion of gas molecule' through the pores during the night cycle when the additional gas supply is inhibited (**Supplementary Fig. 26c**). Thus, J_{ph} was maintained at ~ 4 mA^{-2} over 10 day/night cycles. As evidenced in **Supplementary Fig. 21**, preventing the instant potential change during the night cycle allows stable operation; therefore, maintaining the constant potential during the night cycle could be one plausible solution.

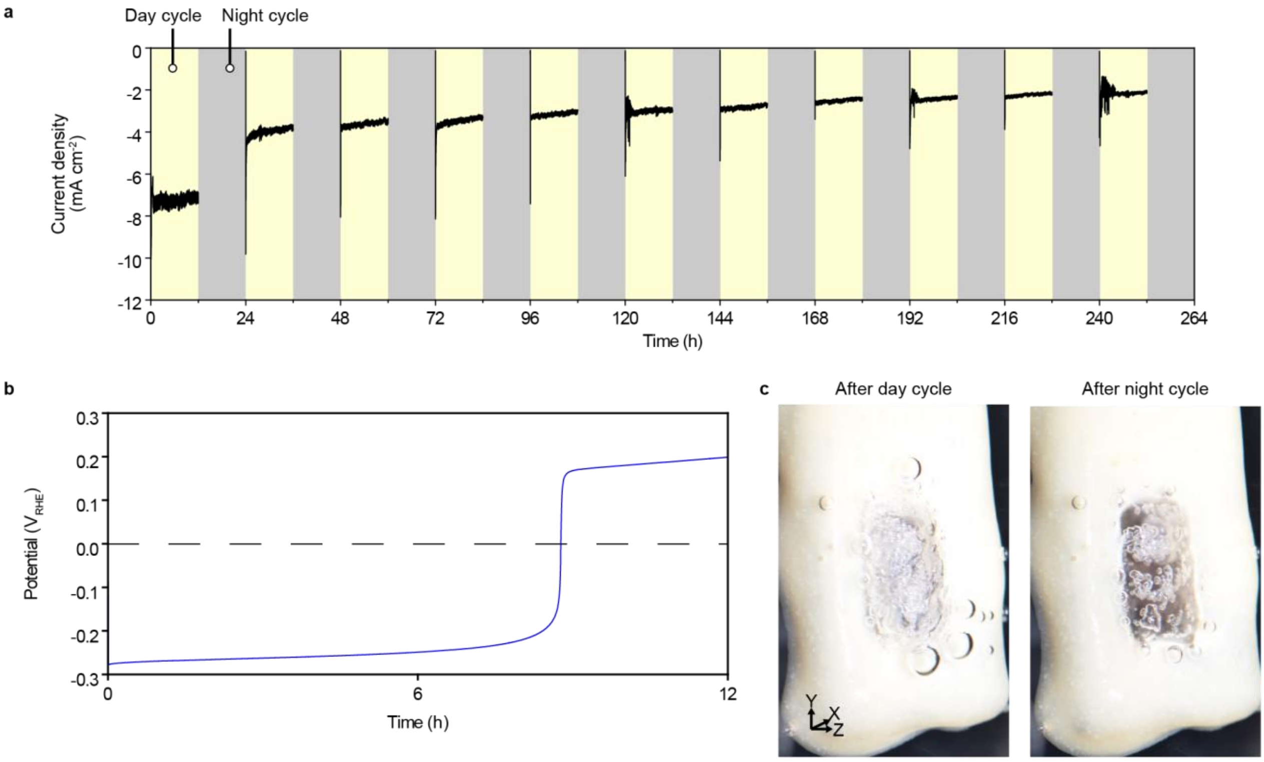

Supplementary Fig. 26. Day/night cycle operation of a Sb_2Se_3 photocathode covered with the cryogelated overlayer with interconnected micropores. (a) The photocurrent density-time profile of the device with the 1200 µm-thick cryogel overlayer *CG-1200µm* operated by day (yellow region) and night (gray region) cycles. The duration of the day and night cycles was 12 h. (b) Open circuit potential of *CG-1200µm* monitored during the first night cycle of 12 h. (c) Picture of the bubbles in *CG-1200µm* after the 'day cycle' and 'night cycle'.

Supplementary Note 4. Characterization of the optical, structural, and electrochemical properties of Pt catalyst.

We fabricated the Pt/fluorine-doped tin oxide **(**FTO**)** electrode using the Pt deposition condition which was the same to those used in the fabrication of the Pt/TiO_2/Sb_2Se_3 photoelectrochemical device. The UV-vis transmittance spectrum of the Pt/FTO electrode normalized by a bare FTO substrate exhibited the optical transmittance of 90% in average (**Supplementary Fig. 27**). The top-view scanning electron microscopy (SEM) images of FTO and Pt/FTO (as-prepared, before electrolysis) were almost identical (**Supplementary Figs. 28a and 28b**), indicating that the Pt nanoparticles were not visible in the SEM image due to its small size. The uniformly deposited Pt nanoparticles with a size of 3-5 nm were found in the transmission electron microscopy image of the cross-sectioned sample of the Pt/FTO electrode (**Supplementary Fig. 28c**).

During the dark electrolysis of Pt/FTO electrode at -0.1 V_{RHE}, the current of the electrode was significantly degraded within 2 h (**Supplementary Fig. 28d**). Since there is no possibility of the photocorrosion of semiconductors for the Pt/FTO electrode, the current degradation of the electrode could be originated from the physical damage of Pt layer. After 2 h of the operation, we observed partial structural damage of the surface of the Pt/FTO device, and the aggregation of Pt particles in some regions (**Supplementary Fig. 28e**).

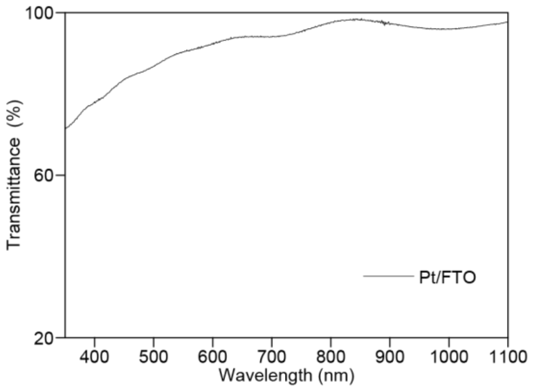

Supplementary Fig. 27. UV-vis transmittance spectrum of the Pt/fluorine-doped tin oxide (FTO) electrode. The bare FTO substrate was utilized as a reference sample.

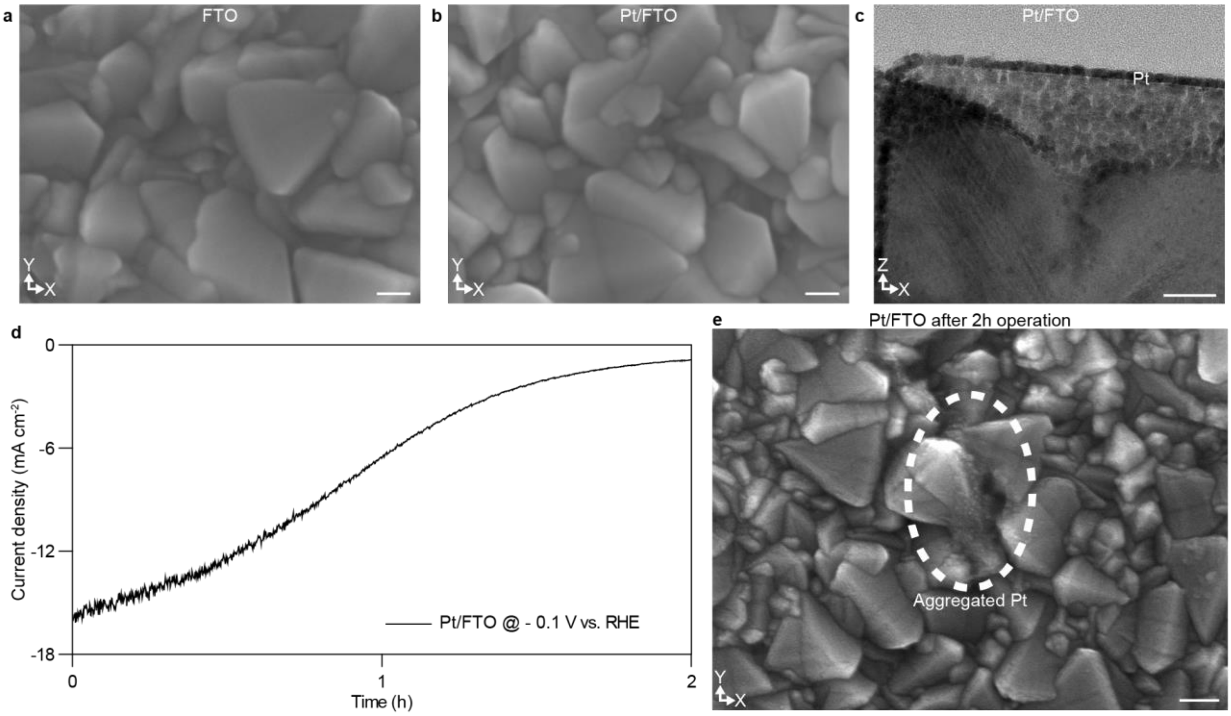

Supplementary Fig. 28. Characterization of the structural damage of Pt catalyst under the strongly cathodic condition. The top-view scanning electron microscopy (SEM) images of (a) fluorine-doped tin oxide (FTO) and (b) Pt/FTO. Scale bars represent 100 nm. (c) The cross-sectional transmission electron microscopy (TEM) image of cross-sectioned Pt/FTO. Scale bar represents 20 nm. (d) Time-current density profile during the chronoamperometry measurement of Pt/FTO. (e) The top-view SEM images of Pt/FTO after 2 h of operation. The white circle represents the region with aggregated Pt catalyst particles. Scale bar represents 200 nm.

Supplementary References

1. Taqieddin, A., Nazari, R., Rajic, L. & Alshawabkeh, A. Review—Physicochemical Hydrodynamics of Gas Bubbles in Two Phase Electrochemical Systems. *Journal of The Electrochemical Society* **164**, E448 (2017).

2. Cho, Y.-J., Yum, S.-B., Lee, J.-H. & Park, G.-C. Development of bubble departure and lift-off diameter models in low heat flux and low flow velocity conditions. *International Journal of Heat and Mass Transfer* **54**, 3234-3244 (2011).

3. Tan, J. *et al.* Hydrogel protection strategy to stabilize water-splitting photoelectrodes. *Nature Energy* **7**, 537-547 (2022).

4. Gautreau, Z., Griffin, J., Peterson, T. & Thongpradit, P. Characterizing viscoelastic properties of polyacrylamide gels. *Worcester, Massachusetts: Worcester Polytechnic Institute* (2006).

5. Yang, W. *et al.* Adjusting the Anisotropy of 1D Sb2Se3 Nanostructures for Highly Efficient Photoelectrochemical Water Splitting. *Advanced Energy Materials* **8**, 1702888 (2018).

6. Paracchino, A., Laporte, V., Sivula, K., Grätzel, M. & Thimsen, E. Highly active oxide photocathode for photoelectrochemical water reduction. *Nature Materials* **10**, 456-461 (2011).

7. Li, C. *et al.* Positive onset potential and stability of Cu2O-based photocathodes in water splitting by atomic layer deposition of a Ga2O3 buffer layer. *Energy & Environmental Science* **8**, 1493-1500 (2015).

8. Septina, W., Prabhakar, R. R., Wick, R., Moehl, T. & Tilley, S. D. Stabilized Solar Hydrogen Production with CuO/CdS Heterojunction Thin Film Photocathodes. *Chemistry of Materials* **29**, 1735-1743 (2017).

9. Yang, W. *et al.* Molecular Chemistry-Controlled Hybrid Ink-Derived Efficient Cu2ZnSnS4 Photocathodes for Photoelectrochemical Water Splitting. *ACS Energy Letters* **1**, 1127-1136 (2016).

10. Ahmed, A. K. A. *et al.* Generation of nanobubbles by ceramic membrane filters: The dependence of bubble size and zeta potential on surface coating, pore size and injected gas pressure. *Chemosphere* **203**, 327-335 (2018).

11. Hutchens, S. B. & Crosby, A. J. Soft-solid deformation mechanics at the tip of an embedded needle. *Soft Matter* **10**, 3679-3684 (2014).

12. Mijailovic, A. S. *et al.* Localized characterization of brain tissue mechanical properties by needle induced cavitation rheology and volume controlled cavity expansion. *J. Mech. Behav. Biomed. Mater.* **114**, 104168 (2021).

13. Ilssar, D. & Gat, A. D. On the inflation and deflation dynamics of liquid-filled, hyperelastic balloons. *Journal of Fluids and Structures* **94**, 102936 (2020).

14. Mitchell, V. Gas, tubes and flow. *Anaesthesia & Intensive Care Medicine* **8**, 7-10 (2007).
